# Resveratrol and the Interaction between Gut Microbiota and Arterial Remodelling

**DOI:** 10.3390/nu12010119

**Published:** 2020-01-01

**Authors:** Andy W.C. Man, Huige Li, Ning Xia

**Affiliations:** Department of Pharmacology, Johannes Gutenberg University Medical Center, 55131 Mainz, Germanyhuigeli@uni-mainz.de (H.L.)

**Keywords:** resveratrol, SIRT1 arterial remodelling, gut microbiota, anti-oxidant, inflammation

## Abstract

Arterial remodelling refers to the alteration in the structure of blood vessel that contributes to the progression of hypertension and other cardiovascular complications. Arterial remodelling is orchestrated by the crosstalk between the endothelium and vascular smooth muscle cells (VSMC). Vascular inflammation participates in arterial remodelling. Resveratrol is a natural polyphenol that possesses anti-oxidant and anti-inflammatory properties and has beneficial effects in both the endothelium and VSMC. Resveratrol has been studied for the protective effects in arterial remodelling and gut microbiota, respectively. Gut microbiota plays a critical role in the immune system and inflammatory processes. Gut microbiota may also regulate vascular remodelling in cardiovascular complications via affecting endothelium function and VSMC proliferation. Currently, there is new evidence showing that gut microbiota regulate the proliferation of VSMC and the formation of neointimal hyperplasia in response to injury. The change in population of the gut microbiota, as well as their metabolites (e.g., short-chain fatty acids) could critically contribute to VSMC proliferation, cell cycle progression, and migration. Recent studies have provided strong evidence that correlate the effects of resveratrol in arterial remodelling and gut microbiota. This review aims to summarize recent findings on the resveratrol effects on cardiovascular complications focusing on arterial remodelling and discuss the possible interactions of resveratrol and the gut microbiota that modulate arterial remodelling.

## 1. Introduction

Cardiovascular disease is the current leading global cause of death and is expected to cause more than 23.6 million deaths by 2030 [1,2]. Hypertension is a strong risk factor for almost all cardiovascular diseases. Hypertension can result from varying factors such as genetics, diet and lifestyle, as well as the gut microbiota [3,4]. Essential hypertension is characterized by the increase of peripheral vascular resistance to blood flow, which generally occurs in small arteries and arterioles that undergo remodelling [5,6]. Arterial remodelling is an active process of structural alteration that includes vascular cell proliferation, migration, death and changes in the extracellular matrix of the artery [6]. The progression of arterial remodelling is modulated by the crosstalk between endothelium and vascular smooth muscle cells (VSMC). The endothelium can sense shear stress generated from blood flow and activate signalling pathways in VSMC [6]. The mechanical effects of blood flow and shear stress in the endothelium and vascular smooth muscles, inflammation, as well as the controls from the renin-angiotensin-aldosterone system, endothelins, adipokines from perivascular adipose tissue (PVAT), are the key factors in the pathophysiology of arterial remodelling and the progression of hypertension [5].

Resveratrol (3,5,4′-trihydroxy-trans-stilbene) is a plant polyphenol phytoalexin found mainly in grape fruits and red wine [7,8]. Resveratrol has anti-oxidant and anti-inflammatory activities [9,10,11]. Massive laboratory and preclinical studies have reported the protective effects of resveratrol in different disease models, including cancer, cardiovascular, metabolic, and neurodegenerative diseases [7,12,13]. Resveratrol is well-studied for its beneficial effects on cardiovascular protection by increasing the production of nitric oxide (NO) in endothelial cells. Resveratrol can upregulate endothelial NO synthase (eNOS) expression, stimulate eNOS activity, and prevent eNOS uncoupling [14]. In addition, resveratrol modulates the function of immune cells, inhibits immune cell infiltration, and improves PVAT function [15,16]. The protective effect of resveratrol against adverse arterial remodelling has been reported recently. Arterial remodelling is an important feature of the progression of hypertension [6,17]. Resveratrol has been studied for its effect on modulating gut microbiota and arterial remodelling, respectively [14,18]. Currently, accumulating evidence has suggested that gut microbiota may participate in the development of metabolic and cardiovascular diseases by interacting with the immune system and inflammatory processes [19]. Targeting the gut microbiota may be an alternative for the treatment of arterial remodelling in cardiovascular diseases, especially in hypertension [20,21]. In this review, we summarize current knowledge of the beneficial effects of resveratrol supplementation in cardiovascular health, focusing on arterial remodelling and gut microbiota and highlight the potential protective effects of resveratrol on arterial remodelling by modulation of gut microbiota population and metabolites.

## 2. Resveratrol and Arterial Remodelling

### 2.1. Improvement of Endothelial Function

Endothelial function plays an important role in regulating arterial remodelling. Removal of the endothelium significantly limits the ability of the vessel to remodel [22]. The shear stress or frictional force generated by the blood flow on the vessel lumen triggers the endothelium to release vasoactive autacoids including NO, prostaglandins, endothelium-derived hyperpolarizing signals and growth factors. These endothelium-derived molecules regulate the ability of the vessel to remodel against different conditions [23,24]. Therefore, the endothelium is the key regulator of blood pressure and vascular tone. Deterioration of endothelial function and smooth muscle tone lead to the stiffening of elastic and muscular arteries [25]. Vasoconstrictors, such as noradrenaline, endothelin-1 (ET-1) or angiotensin II (Ang II), increase artery stiffness [26,27], whereas vasodilators such as glyceryl trinitrate elicit opposite effects [27,28,29,30].

**Figure 1:**
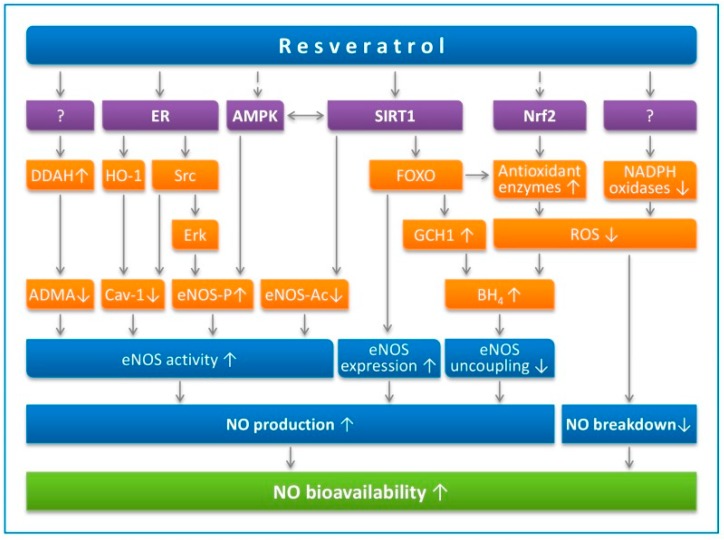
Resveratrol enhances NO production and prevents NO breakdown. Resveratrol activates SIRT1 directly (in a substrate-dependent manner) or indirectly (by either inhibiting phosphodiesterases or enhancing the effect of lamin A). SIRT1 stimulates endothelial NO synthase (eNOS) activity through deacetylation, enhances eNOS expression by deacetylating Forkhead box O (FOXO) transcription factors, and prevents eNOS uncoupling by upregulating GTP cyclohydrolase 1 (GCH1), the rate-limiting enzyme in tetrahydrobiopterin (BH4) biosynthesis. AMP-activated protein kinase (AMPK) and nuclear factor-erythroid-derived 2-related factor-2 (Nrf2) are indirect targets of resveratrol. AMPK phosphorylates eNOS at serine 1177. eNOS can also be phosphorylated by Erk1/2, which is stimulated by a pathway involving estrogen receptors (ER) and the tyrosine kinase Src. Caveolin-1 (Cav-1) is an eNOS-interacting protein that negatively regulates eNOS activity. Asymmetric dimethylarginine (ADMA) is an endogenous eNOS inhibitor that is degraded by dimethylarginine dimethylaminohydrolase (DDAH). The resveratrol targets for DDAH upregulation or for NADPH oxidase downregulation have not been identified so far. Reproduced from Xia et al. *Molecules*. 2014 [36], under the terms of the Creative Commons Attribution-Noncommercial License CC BY-NC.

The congenital absence of eNOS causes adverse vascular remodelling [31,32]. eNOS produces NO which is responsible for the regulation of arterial remodelling [22]. Endothelium-derived NO inhibits growth factor-stimulated proliferation and migration of VSMC [33]. eNOS knockout mice show hyperplasia in the media layer of abnormally remodeled vessels, evidenced by the significant wall thickening, increase in number of nuclei and the incorporation of bromodeoxyuridine. These characteristics are reminiscent of the phenotype of arterial thickening in atherosclerotic and hypertensive patients [34]. The beneficial effects of resveratrol in endothelial function have been widely studied and reported in the aspect of enhancing endothelial NO production, reducing endothelial oxidative stress and ET-1 synthesis (Figure 1) [14,35]. These indicate that resveratrol can target arterial remodelling via enhancing the endothelial NO production.

Sirtuin 1 (SIRT1), which can be activated directly or indirectly by resveratrol, is also known to activate eNOS and enhance endothelial function. Also, resveratrol could improve endothelial function by activating SIRT1 [35]. Activation of SIRT1 by resveratrol is highly controversial. Although resveratrol has been shown to inhibit SIRT1 in some cancer cell models, [37], the beneficial effects of resveratrol in cardiovascular diseases can also be shown in various in vivo experiments which show calorie restriction or SIRT1-overexpression-mimetic effects [38,39]. The exact molecular mechanism of SIRT1 activation by resveratrol remains to be explored, which could be either by parallel or downstream pathways [40].

In endothelial cells, the knocking-down of SIRT1 prevents the resveratrol-induced upregulation and activation of eNOS [41]. Therefore, resveratrol-induced upregulation of eNOS is likely to be SIRT1-dependent. On the other hand, SIRT1/FOXO is involved in the resveratrol-induced eNOS transcriptional activation [42]. In addition to SIRT1/FOXO, endothelial function is also regulated by the SIRT1/Krüpple link factor 2 (KLF2) interaction [43]. In addition, overexpression of endothelial SIRT1 has been shown to prevent adverse arterial remodelling by downregulating LKB1. SIRT1 promotes the protein complex formation between LKB1 and HECT and the RLD domain containing E3 ubiquitin-protein ligase 2 (HERC2) which leads to LKB1 degradation [44]. In clinical studies, the genetic variations of SIRT1 have been reported to be correlated to intimal-medial thickening in human carotid arteries, suggesting that endothelial SIRT1 is important in regulating arterial remodelling [45].

Flow-mediated dilatation (FMD) is commonly used as a noninvasive method to measure the endothelial function in patients [46]. Resveratrol has been shown to reduce blood pressure and improve FMD responses in clinical studies involving patients with metabolic syndromes or hypertension (Table 1). It is conceivable that the improvement of endothelial function and activation of endothelial SIRT1 by resveratrol supplementation are beneficial for preventing arterial remodelling and stiffening.

### 2.2. Inhibition of Neointima Formation

Mature VSMCs are capable of modifying their phenotype in physiological and pathophysiological settings in response to both intrinsic and extrinsic vessel signalling. Normally, differentiated VSMCs have limited synthetic activity, slow proliferation and a specific set of contractile protein expressions [51]. Upon stimulation by various growth factors, or in response to vascular injury, VSMCs show phenotype-switching through proliferation, de-differentiation, and migration to the injury site for vascular repair and remodelling [51]. While this phenotypic plasticity of VSMC is important for the repair and maintenance of the vasculature, excessive activation and proliferation of VSMCs can lead to the development of restenosis, atherosclerosis and hypertension [52]. Also, physical parameters, such as arterial wall pressure, could lead to changes in the contractile state and/or the synthetic activity of VSMCs [53].

Resveratrol has been shown to promote vascular health by maintaining and enhancing the phenotypic plasticity of the VSMC [51,54,55]. Intimal hyperplasia refers to the structural change, including the proliferation and migration of VSMCs from the media and adventitia in the subendothelium with subsequent deposition of significant quantities of extracellular connective tissue [56]. Resveratrol can inhibit VSMC proliferation induced by various pathways including mechanistic Target of Rapamycin (mTOR), protein kinase B (AKT) and 5′ AMP-activated protein kinase (AMPK) [14]. In cultured primary VSMCs, resveratrol has been shown to suppress high glucose-induced oxidative stress and VSMC proliferation by reducing the generation of reactive oxygen species (ROS) and nicotinamide adenine dinucleotide phosphate oxidase (NADPH), downregulating the phosphorylation of Akt/p38 mitogen-activated protein kinase (MAPK)/c-Jun N-terminal kinases (JNK)/extracellular signal–regulated kinases (ERK), and reducing the activities of nuclear factor kappa-light-chain-enhancer of activated B cells (NF-κB) [57]. NF-κB is a major transcription factor participating in inflammatory responses and involving in the initiation and progression of vascular inflammation [58]. Endothelial blockade of intracellular NF-κB signalling markedly suppresses intimal hyperplasia [59]. Resveratrol treatment prevents the proinflammatory properties of the aged VSMC secretome via inhibiting NF-κB [60]. Furthermore, resveratrol has been shown to suppress oxidized low-density lipoprotein (ox-LDL)-induced proliferation of cultured bovine VSMCs [61]. Resveratrol also inhibits parathyroid hormone (PTH)-induced apoptosis in cultured human aortic smooth muscle cells [57].

Neointima formation is associated with the reduced expression of SIRT1, while SIRT1 overexpression in VSMCs prevents neointima formation in response to vascular injury [62]. Also, SIRT1 inhibition increases p53 and plasminogen activator inhibitor-1 (PAI-1) expression, which subsequently leads to injury-induced neointimal formation and remodelling [63]. Resveratrol downregulates Ang II type 1 receptor expression in VSMCs by activating SIRT1 both in vivo and in vitro [64]. Resveratrol also reduces serum Ang II level and prorenin receptor (PRR) and angiotensin-converting enzyme (ACE and ACE2), Ang II type 2 receptor (AT2R), and Mas receptor (MasR) expression in the aorta. Resveratrol can prevent Ang II-induced hypertrophy of VSMCs by normalizing the oxidative stress and activating Proto-oncogene tyrosine-protein kinase (c-Src), growth factor receptors, and MAPK/AKT signalling [65]. Moreover, resveratrol can normalize the ACE, Ang I and II, AT2R, and MasR expression through restoring the expression of SIRT1 in high fat diet (HFD)-fed mice [66] and HFD-fed rats [67]. Therefore, the beneficial effects of resveratrol in neointima formation could be mediated by SIRT1, renin–angiotensin–aldosterone system (RAAS) and NF-κB signalling [68].

In a mice wire-injured arteries model, oral administration of resveratrol significantly suppresses intimal hyperplasia [69,70]. In a rat carotid artery injury model, Intraperitoneal injection of resveratrol inhibits intimal hyperplasia [71]. In addition, periadventitial application of resveratrol has shown significant improvements in intimal hyperplasia, impairment of re-endothelialization and constrictive arterial remodelling, which are the three major pathologies contributing to restenosis [72]. Periadventitial delivery of resveratrol has a greater neointima-inhibiting effect (86%) than systemic resveratrol administration. Moreover, resveratrol promotes post-surgery endothelial recovery without causing constrictive arterial remodelling [72]. These are compelling advantages compared to the current drug-eluting stents used in clinical settings. However, recent study suggests that high concentrations of resveratrol exhibit arginase inhibitory activity in VSMCs that could enhance vasoconstrictor responses [73]. Therefore, the optimal delivery method of resveratrol treatment in arterial remodelling should be further investigated.

### 2.3. Prevention of Arterial Stiffening

Systolic blood pressure is attributed to arterial stiffness which continuously increases with age [74]. Aortic pressure is the instantaneous summation of the reservoir pressure and the effects of the flow wave. Augmented arterial stiffness leads to the increase in blood flow velocity and a backward-travelling reflected wave which further increases systolic pressure [75,76]. Arterial stiffening is a major hallmark of aging and the consequence of many complications such as diabetes, atherosclerosis, and chronic renal diseases [77,78]. Arterial stiffness mainly occurs in the large arteries [6]. Arterial stiffening represents the sum of the passive stiffness, which is mainly contributed to by elastic and collagen fibres, and the active stiffness generated by the smooth muscle tone [79]. Changes in endothelial function and smooth muscle tone can influence the stiffness of the elastic and muscular arteries [25]. Although the stiffening of vasculature is a universal change associated with aging, it is also part of the phenotype in diseases such as hypertension and diabetes where complex cellular mechanisms conspire to accentuate arterial remodelling.

Resveratrol has been shown to prevent arterial remodelling and stiffening in both animal and clinical studies. In rats, both low-dose and high-dose resveratrol treatments improve flow-mediated outward remodelling [80]. A long-term low-dose dietary resveratrol supplement reduces arterial stiffness (measured by the aortic pulse wave velocity) in rats [81]. Surprisingly, resveratrol limits the increase in compliance of spontaneously hypertensive rat (SHR) by its inhibitory effect on arterial remodelling and ERK signalling rather than the effect on blood pressure or arterial wall stiffening [82]. In a recent double blind, randomized, placebo-controlled study, resveratrol supplementation has been shown to reduce arterial stiffness (measured by Cardio Ankle Vascular Index) in patients with type 2 diabetes [47].

In vasculature, collagen and elastin deposition is regulated by matrix metalloproteinases (MMPs) [83]. The latency of MMPs is modulated by eNOS and NO, which can be regulated by resveratrol [84,85,86]. Under pathological conditions, up-regulation of MMPs, as well as the activation of zymogens and infiltration of inflammatory cells lead to arterial remodelling and stiffening [87,88]. Resveratrol has been shown to inhibit MMP expression in various tissues including brain, tumour, as well as VSMCs [89,90,91]. The activity of MMP-2 and MMP-9 is inhibited by resveratrol treatment in different models [90,92,93]. In human VSMCs, tumour necrosis factor alpha (TNF-α)-induced expression of MMP-9 can be inhibited by resveratrol [94]. Upregulation of MMP-2, MMP-9 and their downstream molecule, transforming growth factor-beta 1 (TGF-β1) are responsible for arterial stiffening and blood pressure increase [95]. Therefore, resveratrol could reduce arterial remodelling by regulating the latency of MMPs.

Resveratrol also regulates MMPs and many other inflammatory and pro-oxidative genes associated with remodelling via inhibition of NF-κB signal pathway [15]. Resveratrol inhibits monocrotaline-induced pulmonary arterial remodelling by suppressing sphingosine kinase 1 (SphK1)-mediated NF-κB activation [96]. In H_2_O_2_-treated VSMCs, resveratrol treatment downregulates MMP-9 expression, as well as augmenting the production of tissue inhibitors of metalloproteinases (TIMP-1) [91]. In mice, resveratrol has been shown to prevent high-fat, high-sucrose diet (HFHS)-induced arterial stiffening [97]. Similar results can be obtained with other SIRT1 activators or by global overexpression of SIRT1 [97]. In addition, overnight fasting decreases arterial stiffness acutely in wildtype mice but not in mice with SIRT1-KO in VSMC. Conversely, VSMC-specific SIRT1 overexpression prevents diet-induced arterial stiffness. The anti-remodelling property of SIRT1 is related to its antioxidant and anti-inflammatory effect by inhibiting NF-κB, vascular cell adhesion molecule-1 (VCAM-1) and p47phox expression [97]. These results suggest that inhibition of NF-κB could be a critical mechanism of resveratrol’s beneficial effect in targeting neointima formation and arterial stiffening.

TGF-β1 is an important profibrogenic factor that induces the proliferation of VSMCs and collagen secretion [98,99]. Resveratrol has been demonstrated to block the TGF-β1-stimulated KLF5 production and VSMC de-differentiation [72]. Advanced glycation end-products (AGEs) stimulate non-enzymatic protein glycation forming irreversible cross-links in proteins like collagen. This cross-linking prevents the collagen from regulatory turnover and becomes stiffer [100]. Resveratrol can normalize the AGEs-stimulated TGF-β1 expression and collagen synthesis in cultured rat aortic smooth muscle cells [101]. In addition, overexpression of SIRT1 has been shown to prevent arterial remodelling via reducing TGF-β1-mediated collagen deposition [44]. These results may suggest that resveratrol can target TGF-β1 and NF-κB expression and signalling partly through the activation of SIRT1.

## 3. Gut Microbiota and Microbiota-Derived Metabolites Modulates Arterial Remodelling

### 3.1. Gut Microbiota and Arterial Remodelling

Gut microbiota refers to an ecosystem that consists of numerous species of microbials in the digestive system of the host organism from birth. Gut microbiota is highly interactive with the host and forms a symbiotic signalling mechanism that mutually influences both the host environment and the population of gut microbiota [102]. Currently, gut microbiota is considered a ‘virtual’ endocrine which the microbial metabolites can communicate with distal organs and affect their physiologies and functions. Gut microbiota mainly consists of five phyla—*Bacteroidetes, Firmicutes, Actinobacteria, Proteobacteria* and *Verrucomicrobia.* Different individuals have a distinct abundance and diversity of microbials but the anaerobic *Firmicutes* and *Bacteroidetes* usually occupy more than 90% of the total microbial population [103,104]. The *Firmicutes* to *Bacteroidetes* ratio varies across individuals and the variations are mainly caused by differences in host genomic and environmental factors, such as lifestyle, hygiene status, diet and antibiotic or probiotics treatments [104]. A high *Firmicutes* to *Bacteroidetes* ratio is commonly found to be associated with metabolic and cardiovascular complications [105].

Accumulating evidence has shown that gut microbiota plays an important role in host’s health and diseases [102,106]. Changes in the composition of gut microbiota are linked to the pathology of different cardiovascular complications. In addition to the gut microbiota itself, the microbiota metabolites are also recognized as major contributing factors in the progression of cardiovascular complications. Various clinical and animal studies have provided strong evidence that links specific species to the pathophysiology of different cardiovascular diseases and complications [107]. Nevertheless, the underlying mechanism on how specific bacteria species triggers the progression of cardiovascular diseases is largely unknown.

In recent years, the linkage between gut microbiota and arterial remodelling has become a hot topic. Different animal models have been used to address the association between the gut microbiota and arterial remodelling. In long-term Western diet fed mice, the gut dysbiosis is associated with endothelial dysfunction and arterial stiffening [108]. The observed endothelial dysfunction is correlated with the reduction in the population of *Bifidobacterium spp*. Upon antibiotic treatment, Western diet-induced endothelial dysfunction and arterial stiffening are normalized [108]. In another study, cecal microbiota transplantation from obese mice results in the induction of cardiac ischemic and aortic stiffening in wild type mice. In old mice, antibiotic treatments can reverse endothelial dysfunction and arterial stiffening (measured by pulse wave velocity) accompanied by lower oxidative stress and greater antioxidant enzyme expression [109]. The transplantation of the gut microbiota also associates with increased gut permeability and reduced cecal short-chain fatty acids (SCFA) concentrations [110]. Moreover, after carotid ligation, germ-free mice shows attenuated neointimal hyperplasia development as well as an increased arterial infiltration of anti-inflammatory M2 macrophages and a reduced proportion of mature neutrophils in arteries compared to conventional mouse [111]. These animal studies highlight the importance of healthy gut microbiota in regulating arterial remodelling.

In humans, a recent multivariate analysis shows significant positive associations between VCAM-1 and *Veillonellaceae*, and between ICAM-1 and *Ruminococcus* in obese children, suggesting the interrelationship between endothelial function and gut microbiota [112]. Another recent clinical study suggests that gut microbiome diversity is inversely associated with arterial stiffness in women [21]. A low microbiome diversity correlates with greater arterial stiffness and blood pressure in women. The study also reveals seven operational taxonomic units associated with arterial stiffness (measured by pulse wave velocity) after adjusting for covariates, which includes members of the SCFA-producing *Ruminococcaceae* and *Rikenallaceae* families. While women are more prone to the adverse effects of arterial stiffening including greater augmentation indices and ventricular remodelling [113], this study addresses the relationship between the gut microbiota, arterial remodelling and blood pressure in women. Moreover, aging, a determining factor in arterial remodelling, has been shown to induce critical changes to the population of gut microbiota, such as reduced diversity, a shift in dominant species, increased *Firmicutes* to *Bacteroidetes* ratio, reduced SCFA, and a greater inter-individual variation [114]. Although these studies provide solid evidence that gut microbiome diversity is important in modulating arterial remodelling and stiffening, further studies focusing on a particular microbiota population are needed to design alternative treatments for adverse arterial remodelling.

Gut microbiota has also been shown to exacerbate Ang II-induced arterial hypertension, vascular inflammation and dysfunction in conventional mice compared to germ-free mice [115]. In addition, interleukin-4 (IL-4) and IL-10 are increased in the Ang II-treated conventional mice but not in germ-free mice [115]. However, the authors have not proposed any particular population of gut microbiota responsible for such phenotypes. The translocation of gut bacteria to the intraperitoneal space, due to epithelial layer damage, can induce transitory infection with systemic elevation of IL-12 [116]. IL-12 is shown to be associated with arterial stiffness in healthy individuals [117]. Interestingly, induced pulmonary arterial hypertension in rats also leads to a greater *Firmicutes* to *Bacteroidetes* ratio in the gut microbiota [118]. The gut dysbiosis might play a pathophysiological role in pulmonary arterial hypertension by altering the host immunologic, hormonal and metabolic homeostasis. These studies also suggest a potential relationship between a gut microbial-immune interaction and arterial remodelling.

### 3.2. Gut Microbiota Derived Metabolites and Arterial Remodelling

Targeting the microbiota metabolome may be a valuable alternative for the treatment of adverse arterial remodelling. Gut microbiota is involved in the production of an array of bioactive substances, known as gut microbiota-derived metabolites, contributing to normal physiological function or eliciting diseases [119]. In recent years, different studies have suggested the association between cardiovascular diseases and gut microbiota-derived metabolites [119,120]. While the identification and the modulation of specific population of the gut microbiota could be challenging, the potential treatment interfering the downstream metabolites is possible.

SCFA are saturated fatty acids that are mainly fermented from resistant starch or dietary fibre by gut microbiota [121]. The most common SCFA are acetate, propionate and butyrate. SCFA are generally considered to have beneficial effects on cardiovascular diseases. SCFA inhibit lipopolysaccharide (LPS) or TNFα-induced endothelial inflammatory responses and excessive VCAM-1 expression [122]. SCFA have been shown to reduce blood pressure and arterial stiffness in mice [123]. To date, SCFA are known to contribute to inflammation, gut homeostasis and cardiovascular diseases via binding to G protein-coupled receptors (GPR41, 43, 109A) and vascular olfactory receptor 78 (Olfr78) [122]. Olfr78 is found in olfactory neurons, renal afferent arterioles and in VSMC, where it plays a role in blood pressure regulation [124]. In rats, oral vancomycin treatment exacerbates neointimal hyperplasia development after carotid angioplasty [125]. However, oral supplementation of butyrate can reverse these changes and inhibit VSMC proliferation, migration, and cell cycle progression in a dose-dependent manner in vitro. Butyrate also inhibits superoxide production and consequent Nod-like receptor pyrin domain 3 (Nlrp3) inflammasome formation and activation, which is beneficial against vascular inflammation or intimal hyperplasia [126]. These studies suggest that the association of gut microbial composition with arterial remodelling could potentially occur through an inhibitory effect of butyrate on VSMCs [125].

A recent metagenome-based association study highlights the microbial associations to current and future clinical outcomes related to cardiovascular diseases. The gut microbiota and their interactions with diet and inflammation, including bacterial L-methionine and L-homocysteine biosynthesis, are associated with the incidence and complication of cardiovascular diseases [127]. In rats, methionine diet feeding has been shown to induce carotid arterial remodelling with significantly augmented collagen content [128]. Homocysteinemia (elevated homocysteine level in blood) has been shown to promote the attraction of monocytes and production of proinflammatory cells. Homocysteinemia has been shown to induce arterial remodelling in different mice models [129,130]. Homocysteine is proposed to induce macrophage maturation in arterial walls, as well as vascular inflammation, endothelial dysfunction, VSMC proliferation and oxidative damage with deterioration of arterial wall elastic material [131]. In patients with stable angina, homocysteinemia is associated with coronary artery remodelling [132]. These results suggest that the bacterial L-methionine and L-homocysteine biosynthesis could contribute to homocysteinemia and arterial remodelling.

Trimethylamine-*N*-oxide (TMAO) is generated by a two-step process between the host and gut microbiota. Gut microbiota, especially *Firmicutes* and *Proteobacteria*, converts dietary L-carnitine, choline, and lecithin to trimethylamine by the TMA-generating lyase (CutC/D). Then, the host flavin-containing monoamine oxidases (FMO) are responsible for the conversion to TMAO [133,134]. TMAO is critically involved in the development of atherosclerosis and other cardiovascular diseases. TMAO induces vascular inflammation through MAPK and NF-κB signalling [108]. Plasma TMAO concentration increases with the mortality risk in patients with stable coronary artery disease, as well as the carotid intima-media thickness in obese individuals or patients with thrombosis risk [107,120,135,136,137]. In women, the levels of different gut-derived metabolites, including TMAO, indolepropionate, and phenylacetylglutamine are also associated with arterial stiffening and higher blood pressure [21]. In addition, TMAO level has been shown to increase with age, suggesting a correlation between TMAO and aging-induced arterial remodelling [109,138].

Increased serum indoxylsulfate level is associated with arterial stiffening, aortic calcification and increased mortality in patients with chronic kidney disease [139]. In primary human aortic VSMCs, indoxylsulfate and indoxyl acetate promote thrombosis through upregulating the expression of tissue factor and inhibiting its ubiquitination and degradation, as well as activating the aryl hydrocarbon receptor (AhR) pathway [140]. AhR-deficient mice shows decreased arterial stiffening and a concomitant increase in the activity of eNOS and NO production [141]. These suggest the microbiota-derived indoxylsulfate and indoxyl acetate are associated with arterial remodelling while AhR could be a potential treatment target.

The activation of Toll-like receptor 4 (TLR4) by LPS is involved in the outward carotid arterial remodelling [142], while it also induces the expression of MMP-9 in VSMCs [143]. The binding of LPS to TLR4 activates the downstream pathways including myeloid differentiation protein-88 (MYD88) and NF-κB and contributes to the increased production of pro-inflammatory cytokines such as IL and TNF-α [144,145]. While the relationship between NF-κB and arterial remodelling has been discussed above, MYD88 is also responsible for flow-mediated remodelling via superoxide-initiated inflammation [146]. On the other hand, increased LPS binding protein (LBP) is associated with carotid intima media thickening [147], as well as arterial stiffening [148].

## 4. The Interaction between Resveratrol, Gut Microbiota and Arterial Remodelling

Some reports have argued that the beneficial effects of resveratrol in the cardiovascular system are limited due to the low bioavailability, which may hinder the development as therapeutic agents [149]. However, growing evidence supports the hypothesis that resveratrol is possibly acting through the gut microbiota remodelling [150]. Recently, polyphenol is proposed as potential prebiotics which can shape the gut microbiota composition [151,152]. Shaping the gut microbiota to favour specific species or lowering the *Firmicutes* to *Bacteroidetes* ratio can provide protective effects to the host cardiovascular system [106].

Interestingly, gut microbiota can influence the pathway of resveratrol metabolites production. The gut microbiota is critically involved in the metabolism of resveratrol by increasing its availability from precursors and producing resveratrol-derived metabolites [153]. Resveratrol can be modified by glucuronidation and sulfation in the liver and intestine (Margherita Springer et al. 2019). Piceid (or polydatin) is one of the glucoside forms of resveratrol, which has a higher bioavailability than resveratrol [154]. Unlike resveratrol, which penetrates the cell membrane passively, piceid can enter the cell via an active mechanism using glucose carriers [155]. It has been evidenced that *Bacillus cereus, Bifidobacteria infantis* and *Lactobacillus acidophilus* in the gut microbiota are responsible for the conversion of resveratrol into the piceid [156,157,158]. Interestingly, piceid have similar molecular targets, including SIRT1 and NF-κB, to resveratrol, however, its antioxidant activity is higher than that of resveratrol [153].

On the other hand, gut microbiota can also metabolize resveratrol into certain derivatives including dihydroresveratrol, 3,4′-dihydroxy-trans-stilbene and 3,4′-dihydroxybibenzyl [159]. Normally, high concentrations of dihydroresveratrol and other resveratrol derivatives are detected in plasma and tissues since resveratrol is rapidly metabolized in the body [160]. Currently, there are limited reports on the effects of these resveratrol derivative in relation to metabolic syndrome and cardiovascular diseases. In LPS-treated endothelial cells, 3,4′-dihydroxybibenzyl reduces the expression of pro-inflammatory mediators IL-8 and E-selectin [161]. Interestingly, a recent study suggests sex-related differences in resveratrol metabolism by the gut microbiome [162]. This suggests the importance of the participation of gut microbiota in the modulation of resveratrol effect in targeting arterial remodelling. It would be interesting to elucidate whether these gut microbiota-mediated resveratrol derivatives are able to trigger the beneficial effects in arterial remodelling.

Many studies have addressed the effect of resveratrol on gut microbiota diversity and composition. Resveratrol has been shown to reduce the *Firmicutes* to *Bacteroidetes* ratio, increase the abundances of *Akkermansia,* *Lactobacillus* and *Bifidobacterium* populations and reduce the growth of *Enterococcus faecalis* [163,164,165]. *Enterococcus faecalis* is associated with high levels of extracellular superoxide [166]. A recent study suggests that resveratrol supplementation can increase the population of butyrate producer *Blautia* and *Dorea* in the *Lachnospiraceae* family [167]. Resveratrol also increases the expression of fasting-induced adipose factor (Fiaf), a key gene for triglycerides deposition, which may be associated with the its prebiotic effect on gut microbiota [165]. Sung et al. have demonstrated that high-fat high-salt-fed mice receiving faecal microbiota transplantations from resveratrol treated mice has increased SCFA production [168]. Resveratrol can prevent the TMAO-induced atherosclerosis in ApoE^−/−^ mice, partly due to the decreased TMAO levels via gut microbiota remodelling [163]. Thus, it is speculated that the beneficial effect of resveratrol in arterial remodelling is partly achieved by its ability to alter the gut microbiota diversity.

Recently, intestinal epithelial SIRT1 has been shown to prevent intestinal inflammation by regulating the gut microbiota [169]. The deletion of SIRT1 in the intestinal epithelium results in a reduced abundance of *Bacilli,* particularly *Lactobacillus*, which has anti-inflammatory effect. It is postulated that SIRT1 might be an important mediator of host-microbiome interactions.

### Summary and Future Directions

The protective effects of resveratrol in cardiovascular complications and diseases have been well documented. In future, dissecting the resveratrol effects on modulating both gut microbiota and cardiovascular diseases is crucial. While there are some reports showing resveratrol supplementation results in distinct and even opposite biological effects, one explanation would be the dose-dependent and time-dependent effect of resveratrol. The direction effect of resveratrol in the activation of SIRT1 remains unclear and controversial. It is highly possible that the in vivo action of resveratrol is modulated by the host’s gut microbiota. Recently, Nohr et al. suggest that the anti-inflammatory effect of resveratrol could be attributed to the inhibition of Gram-negative bacteria-derived lipopolysaccharides in the gut [170]. When considering gut microbiota as an important modulator, the controversy between experiment and clinical studies of resveratrol may be explained partly by the individualized gut microbiota population. Therefore, research in resveratrol supplementation should be improved by considering the population of the gut microbiota in the experimental model, especially with the help of advancement of the microbiota sequencing techniques.

To date, the human clinical trials available have shown conflicting or controversial results about the beneficial effects of resveratrol [171]. The use of a humanized (gnotobiotic) rodent model, germ-free animals inoculated with the human faecal microbiota, could be an alternative to address further research questions in the crosstalk between microbiota and resveratrol [172]. So far, no studies have reported the compositional changes of the gut microbiota in humanized rodents treated with resveratrol. The application of metabolomics approaches could identify all gut microbiota metabolites altered by resveratrol treatment and will help to elucidate mechanisms and targets of their activity in arterial remodelling.

**Figure 2:**
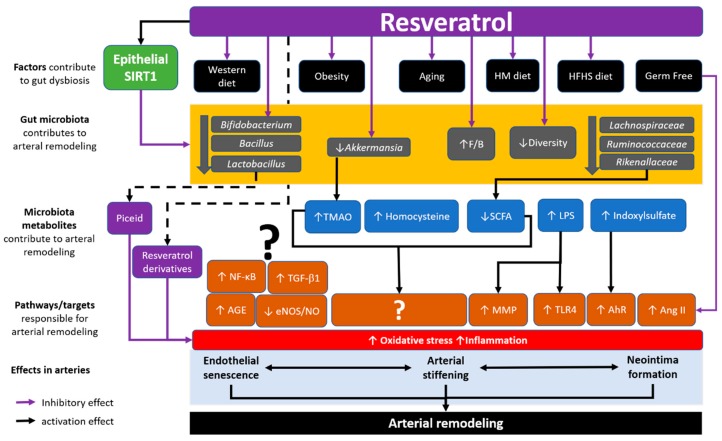
Gut microbiota is a critical player in the effect of resveratrol in arterial remodelling. Environments factors such as diets can affect the population of the gut microbiota. Inappropriate diet such as high fat diet (HFD), as well as aging and obesity, can cause gut dysbiosis. Gut dysbiosis includes the reduction of species diversity, increased F/B ratio and the reduction of good microbes (e.g., *Baillus, Lactobacillus*, *Ruminococcaceae*, *Akkermansia*, etc.) can cause arterial remodelling and other cardiovascular complications. Interestingly, germ-free mice show reduced Ang II activation compared to conventional mice. Resveratrol has been shown to normalize the gut dysbiosis in certain diet models, increase the microbial diversity and good bacteria and reduce the F/B ratio. Resveratrol isoforms and derivatives are modulated by the gut microbiota and have more potent effect in antioxidant and anti-inflammation. Gut dysbiosis also results in the changes in microbiota derived metabolites, including the increase in TMAO, homocysteine, LPS and indoxylsulfate, and the reduction of SCFA (e.g., butyrate). Further studies directions may address the potential pathways and/or targets that are modulated by the microbiota derived metabolites in responsible for arterial remodelling. Also, the association between the known pathways that induce arterial remodelling and gut microbiota and metabolites should be dissected. HM diet: high methionine diet. HFHS diet: high fat high salt diet. F/B: *Firmicutes* to *Bacteroidetes* ratio. TMAO, Trimethylamine-*N*-oxide. LPS, lipopolysaccharide. SCFA, short-chain fatty acids. NF-κB, nuclear factor kappa-light-chain-enhancer of activated B cells. TGF-β1, transforming growth factor-beta 1. AGE, Advanced glycation end-product. eNOS, endothelial nitric oxide synthase. NO, nitric oxide. MMP, matrix metalloproteinases. TLR4, toll-like receptor 4. AhR, aryl hydrocarbon receptor. Ang II, angiotensin II.

Considering the observations made from different studies, we currently hypothesize that gut microbiota is a critical player in the effect of resveratrol on cardiovascular health, especially in arterial remodelling (Figure 2). Resveratrol’s effects on the alteration of the gut microbiota may lead to the change in microbiota-derived metabolites including SCFA, homocysteine, TMAO. However, reports on the direct effect of these metabolites in the cardiovascular system are limited and the underlying mechanisms remain unclear. Further studies should at least include the monitoring of these important microbiota-derived metabolites, as well as to dissect the molecular mechanisms. The translocation of gut microbiota and the induced inflammation can cause arterial remodelling. SIRT1 is an important regulator for cardiovascular health [44,173,174]. The controversy of SIRT1 activation and resveratrol, and the interaction with the gut microbiota, remain an interesting topic that is important in studying the crosstalk between gut microbiota and cardiovascular system.

The scattered evidence from different studies of resveratrol treatment in cardiovascular complications or gut microbiota suggests that the beneficial effects of resveratrol on arterial remodelling and gut microbiota can be linked. This review highlights the importance of a healthy gut microbiota in manifesting the protective effects of resveratrol in cardiovascular disease, especially in arterial remodelling. Supplementation together with specific probiotic may synergize the bioavailability and beneficial effects of resveratrol. Targeting the microbiome may be a valuable alternative for the treatment of arterial remodelling as well as other cardiovascular complications.

## Figures and Tables

**Table 1:** Resveratrol improves endothelial function in metabolic syndromes or hypertension.

Dose and Period	Study Design	Subject Status	Main Findings	Reference
100 mg tablet, oligo-stilbene 27.97 mg/100 mg/day, 12 weeks	25 volunteers; Double blind, randomized, placebo-controlled	T2D	↓ systolic BP; ↓ cardio-ankle vascular index	[47]
ResVida™; 6 capsules, 30, 90, and 270 mg, single dose	19 volunteers; Double blind, randomized, placebo-controlled	Overweight/obese/post-menopausal untreated borderline hypertension	↑ FMD response	[48]
ResVida™; 75 mg capsule/day, 6 weeks	28 obese volunteers; Double blind, randomized, placebo-controlled	Healthy	↑ FMD response; no effect on BP and arterial compliance	[49]
Longevinex, 100 mg/day, 3 months	34 patients with metabolic syndromes; Double blind, randomized, placebo-controlled	Metabolic syndromes and lifestyle-related disease	↑ FMD response; no effect on body composition, lipid profile, interleukin-6 (IL-6) and high-sensitive C-reactive protein (hsCRP).	[50]
300 mg (Bioderm Pharmacy) once daily	24 hypertensive adults; Double blind, cross-over, randomized, placebo-controlled	Hypertension	↑ FMD response; no effect on Augmentation Index, aortic SBP and peripheral BP	[50]

BP: blood pressure. FMD: flow-mediated dilatation. SBP: systolic blood pressure. T2D: type 2 diabetes.

## References

[1] World Health Organization (2012). Cardiovascular Disease: Global Atlas on Cardiovascular Disease Prevention and Control.

[2] Laslett L.J., Alagona P., Clark B.A., Drozda J.P., Saldivar F., Wilson S.R., Poe C., Hart M. (2012). The worldwide environment of cardiovascular disease: Prevalence, diagnosis, therapy, and policy issues: A report from the American College of Cardiology. J. Am. Coll. Cardiol..

[3] Oyama J.-I., Node K. (2019). Gut microbiota and hypertension. Hypertens. Res..

[4] Appel L.J., Brands M.W., Daniels S.R., Karanja N., Elmer P.J., Sacks F.M. (2006). Dietary approaches to prevent and treat hypertension: A scientific statement from the American Heart Association. Hypertension.

[5] Savoia C. (2019). Vascular Remodeling. Textbook of Vascular Medicine.

[6] Man A.W., Wang Y. (2017). Age-Associated Arterial Remodelling. EC Cardiol..

[7] Baur J.A., Sinclair D.A. (2006). Therapeutic potential of resveratrol: the in vivo evidence. Nat. Rev. Drug Discov..

[8] Jang M., Cai L., Udeani G.O., Slowing K.V., Thomas C.F., Beecher C.W., Fong H.H., Farnsworth N.R., Kinghorn A.D., Mehta R.G. (1997). Cancer chemopreventive activity of resveratrol, a natural product derived from grapes. Science.

[9] Bo S., Ciccone G., Castiglione A., Gambino R., De Michieli F., Villois P., Durazzo M., Cavallo-Perin P., Cassader M. (2013). Anti-inflammatory and antioxidant effects of resveratrol in healthy smokers a randomized, double-blind, placebo-controlled, cross-over trial. Curr. Med. Chem..

[10] Peluso I., Villaño Valencia D., Chen C.-Y.O., Palmery M. (2018). Antioxidant, Anti-Inflammatory, and Microbial-Modulating Activities of Nutraceuticals and Functional Foods 2018. Oxid. Med. Cell. Longev..

[11] Aguilar-Alonso P., Vera-López O., Brambila-Colombres E., Segura-Badilla O., Avalos-López R., Lazcano-Hernández M., Navarro-Cruz A. (2018). Evaluation of Oxidative Stress in Cardiomyocytes during the Aging Process in Rats Treated with Resveratrol. Oxid. Med. Cell. Longev..

[12] Li H., Xia N., Förstermann U. (2012). Cardiovascular effects and molecular targets of resveratrol. Nitric Oxide.

[13] Springer M., Moco S. (2019). Resveratrol and Its Human Metabolites—Effects on Metabolic Health and Obesity. Nutrients.

[14] Li H., Xia N., Hasselwander S., Daiber A. (2019). Resveratrol and vascular function. Int. J. Mol. Sci..

[15] Ma C., Wang Y., Dong L., Li M., Cai W. (2015). Anti-inflammatory effect of resveratrol through the suppression of NF-κB and JAK/STAT signaling pathways. Acta Biochim. Biophys. Sin..

[16] Sun Y., Li J., Xiao N., Wang M., Kou J., Qi L., Huang F., Liu B., Liu K. (2014). Pharmacological activation of AMPK ameliorates perivascular adipose/endothelial dysfunction in a manner interdependent on AMPK and SIRT1. Pharmacol. Res..

[17] Gutsol A.A., Blanco P., Samokhina S.I., Afanasiev S.A., Kennedy C.R., Popov S.V., Burns K.D. (2019). A novel method for comparison of arterial remodeling in hypertension: Quantification of arterial trees and recognition of remodeling patterns on histological sections. PLoS ONE.

[18] Chaplin A., Carpéné C., Mercader J. (2018). Resveratrol, metabolic syndrome, and gut microbiota. Nutrients.

[19] Jin M., Qian Z., Yin J., Xu W., Zhou X. (2019). The role of intestinal microbiota in cardiovascular disease. J. Cell. Mol. Med..

[20] Laurent S., Bruno R.M. (2018). Gut microbiome composition, a third player in the inflammation–arterial stiffness relationship. Eur. Heart J..

[21] Menni C., Lin C., Cecelja M., Mangino M., Matey-Hernandez M.L., Keehn L., Mohney R.P., Steves C.J., Spector T.D., Kuo C.-F. (2018). Gut microbial diversity is associated with lower arterial stiffness in women. Eur. Heart J..

[22] Langille B.L., O’Donnell F. (1986). Reductions in arterial diameter produced by chronic decreases in blood flow are endothelium-dependent. Science.

[23] Gibbons G.H., Dzau V.J. (1994). The emerging concept of vascular remodeling. N. Engl. J. Med..

[24] Sandoo A., Veldhuijzen van Zanten J.J., Metsios G.S., Carroll D., Kitas G.D. (2010). The endothelium and its role in regulating vascular tone. Open Cardiovasc. Med. J..

[25] Avolio A., Butlin M., Liu Y.-Y., Viegas K., Avadhanam B., Lindesay G. (2011). Regulation of arterial stiffness: Cellular, molecular and neurogenic mechanisms. Artery Res..

[26] Touyz R.M., Tabet F., Schiffrin E.L. (2003). Redox-dependent signalling by angiotensin II and vascular remodelling in hypertension. Clin. Exp. Pharmacol. Physiol..

[27] Wilkinson I.B., McEniery C.M. (2004). Arterial stiffness, endothelial function and novel pharmacological approaches. Clin. Exp. Pharmacol. Physiol..

[28] Joannides R., Richard V., Haefeli W.E., Benoist A., Linder L., Lüscher T.F., Thuillez C. (1997). Role of nitric oxide in the regulation of the mechanical properties of peripheral conduit arteries in humans. Hypertension.

[29] Wilkinson I.B., MacCallum H., Cockcroft J.R., Webb D.J. (2002). Inhibition of basal nitric oxide synthesis increases aortic augmentation index and pulse wave velocity in vivo. Br. J. Clin. Pharmacol..

[30] Latson T.W., Hunter W.C., Katoh N., Sagawa K. (1988). Effect of nitroglycerin on aortic impedance, diameter, and pulse-wave velocity. Circ. Res..

[31] Kobs R.W., Chesler N.C. (2006). The mechanobiology of pulmonary vascular remodeling in the congenital absence of eNOS. Biomech. Model. Mechanobiol..

[32] Ozaki M., Kawashima S., Yamashita T., Ohashi Y., Rikitake Y., Inoue N., Hirata K.-I., Hayashi Y., Itoh H., Yokoyama M. (2001). Reduced hypoxic pulmonary vascular remodeling by nitric oxide from the endothelium. Hypertension.

[33] Jeremy J.Y., Rowe D., Emsley A.M., Newby A.C. (1999). Nitric oxide and the proliferation of vascular smooth muscle cells. Cardiovasc. Res..

[34] Moroi M., Zhang L., Yasuda T., Virmani R., Gold H.K., Fishman M.C., Huang P.L. (1998). Interaction of genetic deficiency of endothelial nitric oxide, gender, and pregnancy in vascular response to injury in mice. J. Clin. Investig..

[35] Xia N., Daiber A., Förstermann U., Li H. (2017). Antioxidant effects of resveratrol in the cardiovascular system. Br. J. Pharmacol..

[36] Xia N., Förstermann U., Li H. (2014). Resveratrol and endothelial nitric oxide. Molecules.

[37] Buhrmann C., Shayan P., Popper B., Goel A., Shakibaei M. (2016). Sirt1 is required for resveratrol-mediated chemopreventive effects in colorectal cancer cells. Nutrients.

[38] Dang W. (2014). The controversial world of sirtuins. Drug Discov. Today Technol..

[39] Pezzuto J.M. (2019). Resveratrol: Twenty years of growth, development and controversy. Biomol. Ther..

[40] Baur J.A. (2010). Resveratrol, sirtuins, and the promise of a DR mimetic. Mech. Ageing Dev..

[41] Csiszar A., Labinskyy N., Pinto J.T., Ballabh P., Zhang H., Losonczy G., Pearson K., De Cabo R., Pacher P., Zhang C. (2009). Resveratrol induces mitochondrial biogenesis in endothelial cells. Am. J. Physiol. Heart Circ. Physiol..

[42] Xia N., Strand S., Schlufter F., Siuda D., Reifenberg G., Kleinert H., Förstermann U., Li H. (2013). Role of SIRT1 and FOXO factors in eNOS transcriptional activation by resveratrol. Nitric Oxide.

[43] Cui X., Liu X., Feng H., Zhao S., Gao H. (2012). Grape seed proanthocyanidin extracts enhance endothelial nitric oxide synthase expression through 5′-AMP activated protein kinase/Surtuin 1–Krüpple like factor 2 pathway and modulate blood pressure in ouabain induced hypertensive rats. Biol. Pharm. Bull..

[44] Man A.W., Bai B., Yang K., Guo Y., Xu C., Tse H.-F., Han W., Bloksgaard M., De Mey J.G., Vanhoutte P.M. (2016). Endothelial SIRT1 prevents adverse arterial remodeling by facilitating HERC2-mediated degradation of acetylated LKB1. Oncotarget.

[45] Kedenko L., Lamina C., Kedenko I., Kollerits B., Kiesslich T., Iglseder B., Kronenberg F., Paulweber B. (2014). Genetic polymorphisms at SIRT1 and FOXO1 are associated with carotid atherosclerosis in the SAPHIR cohort. BMC Med. Genet..

[46] Faulx M.D., Wright A.T., Hoit B.D. (2003). Detection of endothelial dysfunction with brachial artery ultrasound scanning. Am. Heart J..

[47] Imamura H., Yamaguchi T., Nagayama D., Saiki A., Shirai K., Tatsuno I. (2017). Resveratrol ameliorates arterial stiffness assessed by cardio-ankle vascular index in patients with type 2 diabetes mellitus. Int. Heart J..

[48] Wong R., Howe P., Buckley J., Coates A., Kunz I., Berry N. (2011). Acute resveratrol supplementation improves flow-mediated dilatation in overweight/obese individuals with mildly elevated blood pressure. Nutr. Metab. Cardiovasc. Dis..

[49] Wong R.H., Berry N.M., Coates A.M., Buckley J.D., Bryan J., Kunz I., Howe P.R. (2013). Chronic resveratrol consumption improves brachial flow-mediated dilatation in healthy obese adults. J. Hypertens..

[50] Fujitaka K., Otani H., Jo F., Jo H., Nomura E., Iwasaki M., Nishikawa M., Iwasaka T., Das D.K. (2011). Modified resveratrol Longevinex improves endothelial function in adults with metabolic syndrome receiving standard treatment. Nutr. Res..

[51] Thompson A.M., Martin K.A., Rzucidlo E.M. (2014). Resveratrol induces vascular smooth muscle cell differentiation through stimulation of SirT1 and AMPK. PLoS ONE.

[52] Wang D., Uhrin P., Mocan A., Waltenberger B., Breuss J.M., Tewari D., Mihaly-Bison J., Huminiecki Ł., Starzyński R.R., Tzvetkov N.T. (2018). Vascular smooth muscle cell proliferation as a therapeutic target. Part 1: Molecular targets and pathways. Biotechnol. Adv..

[53] Kamiya A., Togawa T. (1980). Adaptive regulation of wall shear stress to flow change in the canine carotid artery. Am. J. Physiol. Heart Circ. Physiol..

[54] Ong E.-T., Hwang T.-L., Huang Y.-L., Lin C.-F., Wu W.-B. (2011). Vitisin B, a resveratrol tetramer, inhibits migration through inhibition of PDGF signaling and enhancement of cell adhesiveness in cultured vascular smooth muscle cells. Toxicol. Appl. Pharmacol..

[55] Ekshyyan V.P., Hebert V.Y., Khandelwal A., Dugas T.R. (2007). Resveratrol inhibits rat aortic vascular smooth muscle cell proliferation via estrogen receptor dependent nitric oxide production. J. Cardiovasc. Pharmacol..

[56] Heckenkamp J., Lamuraglia G.M. (1999). Intimal hyperplasia, arterial remodeling, and restenosis: An overview. Perspect. Vasc. Surg. Endovasc. Ther..

[57] Guo R., Li W., Liu B., Li S., Zhang B., Xu Y. (2014). Resveratrol protects vascular smooth muscle cells against high glucose-induced oxidative stress and cell proliferation in vitro. Med. Sci. Monit. Basic Res..

[58] Xia N., Forstermann U., Li H. (2014). Resveratrol as a gene regulator in the vasculature. Curr. Pharm. Biotechnol..

[59] Saito T., Hasegawa Y., Ishigaki Y., Yamada T., Gao J., Imai J., Uno K., Kaneko K., Ogihara T., Shimosawa T. (2012). Importance of endothelial NF-κB signalling in vascular remodelling and aortic aneurysm formation. Cardiovasc. Res..

[60] Csiszar A., Sosnowska D., Wang M., Lakatta E.G., Sonntag W.E., Ungvari Z. (2012). Age-associated proinflammatory secretory phenotype in vascular smooth muscle cells from the non-human primate Macaca mulatta: Reversal by resveratrol treatment. J. Gerontol. Ser. A Biomed. Sci. Med. Sci..

[61] Liu Y., Liu G. (2004). Isorhapontigenin and resveratrol suppress oxLDL-induced proliferation and activation of ERK1/2 mitogen-activated protein kinases of bovine aortic smooth muscle cells. Biochem. Pharmacol..

[62] Li L., Zhang H.-N., Chen H.-Z., Gao P., Zhu L.-H., Li H.-L., Lv X., Zhang Q.-J., Zhang R., Wang Z. (2011). SIRT1 acts as a modulator of neointima formation following vascular injury in mice. Circ. Res..

[63] Wan Y.Z., Gao P., Zhou S., Zhang Z.Q., Hao D.L., Lian L.S., Li Y.J., Chen H.Z., Liu D.P. (2014). SIRT1-mediated epigenetic downregulation of plasminogen activeator inhibitor-1 prevents vascular endothelial replicative senescence. Aging Cell.

[64] Miyazaki R., Ichiki T., Hashimoto T., Inanaga K., Imayama I., Sadoshima J., Sunagawa K. (2008). SIRT1, a longevity gene, downregulates angiotensin II type 1 receptor expression in vascular smooth muscle cells. Arterioscler. Thromb. Vasc. Biol..

[65] Hossain E., Anand-Srivastava M.B. (2017). Resveratrol prevents angiotensin II-induced hypertrophy of vascular smooth muscle cells through the transactivation of growth factor receptors. Can. J. Physiol. Pharmacol..

[66] Sheen J.-M., Yu H.-R., Tain Y.-L., Tsai W.-L., Tiao M.-M., Lin I.-C., Tsai C.-C., Lin Y.-J., Huang L.-T. (2018). Combined maternal and postnatal high-fat diet leads to metabolic syndrome and is effectively reversed by resveratrol: A multiple-organ study. Sci. Rep..

[67] Tiao M.-M., Lin Y.-J., Yu H.-R., Sheen J.-M., Lin I.-C., Lai Y.-J., Tain Y.-L., Huang L.-T., Tsai C.-C. (2018). Resveratrol ameliorates maternal and post-weaning high-fat diet-induced nonalcoholic fatty liver disease via renin-angiotensin system. Lipids Health Dis..

[68] Jang I., Kim E., Lim J., Kim M., Ban T., Yoon H., Park C., Chang Y., Choi B. (2018). Effects of Resveratrol on the Renin-Angiotensin System in the Aging Kidney. Nutrients.

[69] Kim J.W., Lim S.C., Lee M.Y., Lee J.W., Oh W.K., Kim S.K., Kang K.W. (2010). Inhibition of neointimal formation by trans-resveratrol: Role of phosphatidyl inositol 3-kinase-dependent Nrf2 activation in heme oxygenase-1 induction. Mol. Nutr. Food Res..

[70] Khandelwal A.R., Hebert V.Y., Kleinedler J.J., Rogers L.K., Ullevig S.L., Asmis R., Shi R., Dugas T.R. (2012). Resveratrol and quercetin interact to inhibit neointimal hyperplasia in mice with a carotid injury. J. Nutr..

[71] Orozco-Sevilla V., Naftalovich R., Hoffmann T., London D., Czernizer E., Yang C., Dardik A., Dardik H. (2013). Epigallocatechin-3-gallate is a potent phytochemical inhibitor of intimal hyperplasia in the wire-injured carotid artery. J. Vasc. Surg..

[72] Zhu Y., Takayama T., Wang B., Kent A., Zhang M., Binder B.Y., Urabe G., Shi Y., DiRenzo D., Goel S.A. (2017). Restenosis Inhibition and Re-differentiation of TGFβ/Smad3-activated Smooth Muscle Cells by Resveratrol. Sci. Rep..

[73] Choi C.I., Koo B.H., Hong D., Kwon H.J., Hoe K.L., Won M.H., Kim Y.M., Lim H.K., Ryoo S. (2019). Resveratrol is an arginase inhibitor contributing to vascular smooth muscle cell vasoconstriction via increasing cytosolic calcium. Mol. Med. Rep..

[74] Dyck G.J., Raj P., Zieroth S., Dyck J.R., Ezekowitz J.A. (2019). The Effects of Resveratrol in Patients with Cardiovascular Disease and Heart Failure: A Narrative Review. Int. J. Mol. Sci..

[75] Oliver J.J., Webb D.J. (2003). Noninvasive assessment of arterial stiffness and risk of atherosclerotic events. Arterioscler. Thromb. Vasc. Biol..

[76] Wang J.-J., O’Brien A.B., Shrive N.G., Parker K.H., Tyberg J.V. (2003). Time-domain representation of ventricular-arterial coupling as a windkessel and wave system. Am. J. Physiol. Heart Circ. Physiol..

[77] Lakatta E.G. (2003). Arterial and cardiac aging: Major shareholders in cardiovascular disease enterprises. Circulation.

[78] Roy C.S. (1881). The elastic properties of the arterial wall. J. Physiol..

[79] Lacolley P., Challande P., Osborne-Pellegrin M., Regnault V. (2008). Genetics and pathophysiology of arterial stiffness. Cardiovasc. Res..

[80] Petit M., Guihot A.-L., Grimaud L., Vessieres E., Toutain B., Menet M.-C., Nivet-Antoine V., Arnal J.-F., Loufrani L., Procaccio V. (2016). Resveratrol improved flow-mediated outward arterial remodeling in ovariectomized rats with hypertrophic effect at high dose. PLoS ONE.

[81] Ahmet I., Tae H.-J., Lakatta E.G., Talan M. (2016). Long-term low dose dietary resveratrol supplement reduces cardiovascular structural and functional deterioration in chronic heart failure in rats. Can. J. Physiol. Pharmacol..

[82] Behbahani J., Thandapilly S.J., Louis X.L., Huang Y., Shao Z., Kopilas M.A., Wojciechowski P., Netticadan T., Anderson H.D. (2010). Resveratrol and small artery compliance and remodeling in the spontaneously hypertensive rat. Am. J. Hypertens..

[83] Jacob M.P. (2003). Extracellular matrix remodeling and matrix metalloproteinases in the vascular wall during aging and in pathological conditions. Biomed. Pharmacother..

[84] Zaragoza C., Balbín M., López-Otín C., Lamas S. (2002). Nitric oxide regulates matrix metalloprotease-13 expression and activity in endothelium. Kidney Int..

[85] Upchurch G.R., Ford J.W., Weiss S.J., Knipp B.S., Peterson D.A., Thompson R.W., Eagleton M.J., Broady A.J., Proctor M.C., Stanley J.C. (2001). Nitric oxide inhibition increases matrix metalloproteinase–9 expression by rat aortic smooth muscle cells in vitro. J. Vasc. Surg..

[86] Tronc F., Mallat Z., Lehoux S., Wassef M., Esposito B., Tedgui A. (2000). Role of Matrix Metalloproteinases in Blood Flow–Induced Arterial Enlargement. Arterioscler. Thromb. Vasc. Biol..

[87] Lu P., Takai K., Weaver V.M., Werb Z. (2011). Extracellular matrix degradation and remodeling in development and disease. Cold Spring Harb. Perspect. Biol..

[88] O’Sullivan S., Medina C., Ledwidge M., Radomski M.W., Gilmer J.F. (2014). Nitric oxide-matrix metaloproteinase-9 interactions: Biological and pharmacological significance--NO and MMP-9 interactions. Biochim. Biophys. Acta.

[89] Bai Y., Yang H., Zhang G., Hu L., Lei Y., Qin Y., Yang Y., Wang Q., Li R., Mao Q. (2017). Inhibitory effects of resveratrol on the adhesion, migration and invasion of human bladder cancer cells. Mol. Med. Rep..

[90] Pandey A.K., Bhattacharya P., Swet Chand Shukla S.P., Patnaik R. (2015). Resveratrol inhibits matrix metalloproteinases to attenuate neuronal damage in cerebral ischemia: A molecular docking study exploring possible neuroprotection. Neural Regen. Res..

[91] Farrokhi E., Ghatreh-Samani K., Salehi-Vanani N., Mahmoodi A. (2018). The effect of resveratrol on expression of matrix metalloproteinase 9 and its tissue inhibitors in vascular smooth muscle cells. ARYA Atheroscler..

[92] Gagliano N., Moscheni C., Torri C., Magnani I., Bertelli A.A., Gioia M. (2005). Effect of resveratrol on matrix metalloproteinase-2 (MMP-2) and Secreted Protein Acidic and Rich in Cysteine (SPARC) on human cultured glioblastoma cells. Biomed. Pharmacother..

[93] Gweon E.J., Kim S.J. (2013). Resveratrol induces MMP-9 and cell migration via the p38 kinase and PI-3K pathways in HT1080 human fibrosarcoma cells. Oncol. Rep..

[94] Lee B., Moon S.-K. (2005). Resveratrol Inhibits TNF-α–Induced Proliferation and Matrix Metalloproteinase Expression in Human Vascular Smooth Muscle Cells. J. Nutr..

[95] Wang M., Kim S.H., Monticone R.E., Lakatta E.G. (2015). Matrix metalloproteinases promote arterial remodeling in aging, hypertension, and atherosclerosis. Hypertension.

[96] Shi W., Zhai C., Feng W., Wang J., Zhu Y., Li S., Wang Q., Zhang Q., Yan X., Chai L. (2018). Resveratrol inhibits monocrotaline-induced pulmonary arterial remodeling by suppression of SphK1-mediated NF-κB activation. Life Sci..

[97] Fry J.L., Al Sayah L., Weisbrod R.M., Van Roy I., Weng X., Cohen R.A., Bachschmid M.M., Seta F. (2016). Vascular smooth muscle sirtuin-1 protects against diet-induced aortic stiffness. Hypertension.

[98] Zhu W., Kim B.C., Wang M., Huang J., Isak A., Bexiga N.M., Monticone R., Ha T., Lakatta E.G., An S.S. (2018). TGFβ1 reinforces arterial aging in the vascular smooth muscle cell through a long-range regulation of the cytoskeletal stiffness. Sci. Rep..

[99] Rensen S., Doevendans P., Van Eys G. (2007). Regulation and characteristics of vascular smooth muscle cell phenotypic diversity. Neth. Heart J..

[100] Bailey A.J. (2001). Molecular mechanisms of ageing in connective tissues. Mech. Ageing Dev..

[101] Mizutani K., Ikeda K., Yamori Y. (2000). Resveratrol inhibits AGEs-induced proliferation and collagen synthesis activity in vascular smooth muscle cells from stroke-prone spontaneously hypertensive rats. Biochem. Biophys. Res. Commun..

[102] Cardona F., Andrés-Lacueva C., Tulipani S., Tinahones F.J., Queipo-Ortuño M.I. (2013). Benefits of polyphenols on gut microbiota and implications in human health. J. Nutr. Biochem..

[103] Castaner O., Goday A., Park Y.-M., Lee S.-H., Magkos F., Shiow S.-A.T.E., Schröder H. (2018). The gut microbiome profile in obesity: A systematic review. Int. J. Endocrinol..

[104] Qin J., Li R., Raes J., Arumugam M., Burgdorf K.S., Manichanh C., Nielsen T., Pons N., Levenez F., Yamada T. (2010). A human gut microbial gene catalogue established by metagenomic sequencing. Nature.

[105] Ley R.E., Bäckhed F., Turnbaugh P., Lozupone C.A., Knight R.D., Gordon J.I. (2005). Obesity alters gut microbial ecology. Proc. Natl. Acad. Sci. USA.

[106] Espín J.C., González-Sarrías A., Tomás-Barberán F.A. (2017). The gut microbiota: A key factor in the therapeutic effects of (poly) phenols. Biochem. Pharmacol..

[107] Kiouptsi K., Reinhardt C. (2018). Contribution of the commensal microbiota to atherosclerosis and arterial thrombosis. Br. J. Pharmacol..

[108] De Bruyne T., Steenput B., Roth L., De Meyer G.R.Y., dos Santos C.N., Valentová K., Dambrova M., Hermans N. (2019). Dietary Polyphenols Targeting Arterial Stiffness: Interplay of Contributing Mechanisms and Gut Microbiome-Related Metabolism. Nutrients.

[109] Brunt V.E., Gioscia-Ryan R.A., Richey J.J., Zigler M.C., Cuevas L.M., Gonzalez A., Vázquez-Baeza Y., Battson M.L., Smithson A.T., Gilley A.D. (2019). Suppression of the gut microbiome ameliorates age-related arterial dysfunction and oxidative stress in mice. J. Physiol..

[110] Battson M.L., Lee D.M., Li Puma L.C., Ecton K.E., Thomas K.N., Febvre H.P., Chicco A.J., Weir T.L., Gentile C.L. (2019). Gut microbiota regulates cardiac ischemic tolerance and aortic stiffness in obesity. Am. J. Physiol. Heart Circ. Physiol..

[111] Wun K., Theriault B.R., Pierre J.F., Chen E.B., Leone V.A., Harris K.G., Xiong L., Jiang Q., Spedale M., Eskandari O.M. (2018). Microbiota control acute arterial inflammation and neointimal hyperplasia development after arterial injury. PLoS ONE.

[112] Nirmalkar K., Murugesan S., Pizano-Zárate M., Villalobos-Flores L., García-González C., Morales-Hernández R., Nuñez-Hernández J., Hernández-Quiroz F., Romero-Figueroa M., Hernández-Guerrero C. (2018). Gut Microbiota and Endothelial Dysfunction Markers in Obese Mexican Children and Adolescents. Nutrients.

[113] Beale A.L., Kaye D.M., Marques F.Z. (2019). The role of the gut microbiome in sex differences in arterial pressure. Biol. Sex Differ..

[114] Valdés L., Cuervo A., Salazar N., Ruas-Madiedo P., Gueimonde M., González S. (2015). The relationship between phenolic compounds from diet and microbiota: Impact on human health. Food Funct..

[115] Karbach S.H., Schönfelder T., Brandão I., Wilms E., Hörmann N., Jäckel S., Schüler R., Finger S., Knorr M., Lagrange J. (2016). Gut microbiota promote angiotensin II–induced arterial hypertension and vascular dysfunction. J. Am. Heart Assoc..

[116] Paulos C.M., Wrzesinski C., Kaiser A., Hinrichs C.S., Chieppa M., Cassard L., Palmer D.C., Boni A., Muranski P., Yu Z. (2007). Microbial translocation augments the function of adoptively transferred self/tumor-specific CD8+ T cells via TLR4 signaling. J. Clin. Investig..

[117] Yong K., Dogra G., Boudville N., Chan D., Adams L., Ching H., Lim E.M., Lim W.H. (2013). Interleukin-12 is associated with arterial stiffness in healthy individuals. Am. J. Hypertens..

[118] Callejo M., Mondejar-Parreño G., Barreira B., Izquierdo-Garcia J.L., Morales-Cano D., Esquivel-Ruiz S., Moreno L., Cogolludo Á., Duarte J., Perez-Vizcaino F. (2018). Pulmonary arterial hypertension affects the rat gut microbiome. Sci. Rep..

[119] Wang Z., Zhao Y. (2018). Gut microbiota derived metabolites in cardiovascular health and disease. Protein Cell.

[120] Wang Z., Klipfell E., Bennett B.J., Koeth R., Levison B.S., DuGar B., Feldstein A.E., Britt E.B., Fu X., Chung Y.-M. (2011). Gut flora metabolism of phosphatidylcholine promotes cardiovascular disease. Nature.

[121] Kau A.L., Ahern P.P., Griffin N.W., Goodman A.L., Gordon J.I. (2011). Human nutrition, the gut microbiome and the immune system. Nature.

[122] Li M., van Esch B.C., Henricks P.A., Folkerts G., Garssen J. (2018). The anti-inflammatory effects of short chain fatty acids on lipopolysaccharide-or tumor necrosis factor α-stimulated endothelial cells via activation of GPR41/43 and inhibition of HDACs. Front. Pharmacol..

[123] Natarajan N., Hori D., Flavahan S., Steppan J., Flavahan N.A., Berkowitz D.E., Pluznick J.L. (2016). Microbial short chain fatty acid metabolites lower blood pressure via endothelial G protein-coupled receptor 41. Physiol. Genom..

[124] Pluznick J.L., Protzko R.J., Gevorgyan H., Peterlin Z., Sipos A., Han J., Brunet I., Wan L.-X., Rey F., Wang T. (2013). Olfactory receptor responding to gut microbiota-derived signals plays a role in renin secretion and blood pressure regulation. Proc. Natl. Acad. Sci..

[125] Ho K.J., Xiong L., Hubert N.J., Nadimpalli A., Wun K., Chang E.B., Kibbe M.R. (2015). Vancomycin treatment and butyrate supplementation modulate gut microbe composition and severity of neointimal hyperplasia after arterial injury. Physiol. Rep..

[126] Yuan X., Wang L., Bhat O.M., Lohner H., Li P.-L. (2018). Differential effects of short chain fatty acids on endothelial Nlrp3 inflammasome activation and neointima formation: Antioxidant action of butyrate. Redox Biol..

[127] Kurilshikov A., van den Munckhof I.C., Chen L., Bonder M.J., Schraa K., Rutten J.H., Riksen N.P., de Graaf J., Oosting M., Sanna S. (2019). Gut Microbial Associations to Plasma Metabolites Linked to Cardiovascular Phenotypes and Risk: A Cross-Sectional Study. Circ. Res..

[128] Huo Y., Wu X., Ding J., Geng Y., Qiao W., Ge A., Guo C., Lv J., Bao H., Fan W. (2018). Vascular Remodeling, Oxidative Stress, and Disrupted PPARγ Expression in Rats of Long-Term Hyperhomocysteinemia with Metabolic Disturbance. PPAR Res..

[129] Kumar M., Tyagi N., Moshal K.S., Sen U., Kundu S., Mishra P.K., Givvimani S., Tyagi S.C. (2008). Homocysteine decreases blood flow to the brain due to vascular resistance in carotid artery. Neurochem. Int..

[130] Munjal C., Givvimani S., Qipshidze N., Tyagi N., Falcone J., Tyagi S. (2011). Mesenteric vascular remodeling in hyperhomocysteinemia. Mol. Cell. Biochem..

[131] Zhang J.-X., Wang Z.-M., Zhang J.-J., Zhu L.-L., Gao X.-F., Chen S.-L. (2014). Association of glutathione peroxidase-1 (GPx-1) rs1050450 Pro198Leu and Pro197Leu polymorphisms with cardiovascular risk: A meta-analysis of observational studies. J. Geriatr. Cardiol. JGC.

[132] Hong M.K., Park S.W., Lee C.W., Choi S.W., Song J.M., Kang D.H., Song J.K., Kim J.J., Park S.J. (2002). Elevated homocysteine levels might be associated with coronary artery remodeling in patients with stable angina: An intravascular ultrasound study. Clin. Cardiol. Int. Index. Peer Rev. J. Adv. Treat. Cardiovasc. Dis..

[133] Velasquez M., Ramezani A., Manal A., Raj D. (2016). Trimethylamine N-oxide: the good, the bad and the unknown. Toxins.

[134] Rath S., Heidrich B., Pieper D.H., Vital M. (2017). Uncovering the trimethylamine-producing bacteria of the human gut microbiota. Microbiome.

[135] Senthong V., Wang Z., Li X.S., Fan Y., Wu Y., Wilson Tang W., Hazen S.L. (2016). Intestinal microbiota-generated metabolite trimethylamine-N-oxide and 5-year mortality risk in stable coronary artery disease: The contributory role of intestinal microbiota in a COURAGE-like patient cohort. J. Am. Heart Assoc..

[136] Randrianarisoa E., Lehn-Stefan A., Wang X., Hoene M., Peter A., Heinzmann S.S., Zhao X., Königsrainer I., Königsrainer A., Balletshofer B. (2016). Relationship of serum trimethylamine N-oxide (TMAO) levels with early atherosclerosis in humans. Sci. Rep..

[137] Zhu W., Gregory J.C., Org E., Buffa J.A., Gupta N., Wang Z., Li L., Fu X., Wu Y., Mehrabian M. (2016). Gut microbial metabolite TMAO enhances platelet hyperreactivity and thrombosis risk. Cell.

[138] Li T., Chen Y., Gua C., Li X. (2017). Elevated circulating trimethylamine N-oxide levels contribute to endothelial dysfunction in aged rats through vascular inflammation and oxidative stress. Front. Physiol..

[139] Barreto F.C., Barreto D.V., Liabeuf S., Meert N., Glorieux G., Temmar M., Choukroun G., Vanholder R., Massy Z.A., Group EUTWG (2009). Serum indoxyl sulfate is associated with vascular disease and mortality in chronic kidney disease patients. Clin. J. Am. Soc. Nephrol..

[140] Chitalia V.C., Shivanna S., Martorell J., Balcells M., Bosch I., Kolandaivelu K., Edelman E.R. (2013). Uremic serum and solutes increase post–vascular interventional thrombotic risk through altered stability of smooth muscle cell tissue factor. Circulation.

[141] Eckers A., Jakob S., Heiss C., Haarmann-Stemmann T., Goy C., Brinkmann V., Cortese-Krott M.M., Sansone R., Esser C., Ale-Agha N. (2016). The aryl hydrocarbon receptor promotes aging phenotypes across species. Sci. Rep..

[142] Hollestelle S.C., de Vries M.R., van Keulen J.K., Schoneveld A.H., Vink A., Strijder C.F., van Middelaar B.J., Pasterkamp G., Quax P.H., de Kleijn D.P. (2004). Toll-like receptor 4 is involved in outward arterial remodeling. Circulation.

[143] Li H., Xu H., Liu S. (2011). Toll-like receptors 4 induces expression of matrix metalloproteinase-9 in human aortic smooth muscle cells. Mol. Biol. Rep..

[144] Ma J., Li H. (2018). The role of gut microbiota in atherosclerosis and hypertension. Front. Pharmacol..

[145] Li H., Xu H., Sun B. (2012). Lipopolysaccharide regulates MMP-9 expression through TLR4/NF-κB signaling in human arterial smooth muscle cells. Mol. Med. Rep..

[146] Tang P.C., Qin L., Zielonka J., Zhou J., Matte-Martone C., Bergaya S., van Rooijen N., Shlomchik W.D., Min W., Sessa W.C. (2008). MyD88-dependent, superoxide-initiated inflammation is necessary for flow-mediated inward remodeling of conduit arteries. J. Exp. Med..

[147] Serrano M., Moreno-Navarrete J.M., Puig J., Moreno M., Guerra E., Ortega F., Xifra G., Ricart W., Fernández-Real J.M. (2013). Serum lipopolysaccharide-binding protein as a marker of atherosclerosis. Atherosclerosis.

[148] Sakura T., Morioka T., Shioi A., Kakutani Y., Miki Y., Yamazaki Y., Motoyama K., Mori K., Fukumoto S., Shoji T. (2017). Lipopolysaccharide-binding protein is associated with arterial stiffness in patients with type 2 diabetes: A cross-sectional study. Cardiovasc. Diabetol..

[149] Subramanian L., Youssef S., Bhattacharya S., Kenealey J., Polans A.S., van Ginkel P.R. (2010). Resveratrol: Challenges in translation to the clinic—A critical discussion. Clin. Cancer Res..

[150] Anhê F.F., Roy D., Pilon G., Dudonné S., Matamoros S., Varin T.V., Garofalo C., Moine Q., Desjardins Y., Levy E. (2015). A polyphenol-rich cranberry extract protects from diet-induced obesity, insulin resistance and intestinal inflammation in association with increased Akkermansia spp. population in the gut microbiota of mice. Gut.

[151] Etxeberria U., Fernández-Quintela A., Milagro F.I., Aguirre L., Martínez J.A., Portillo M.P. (2013). Impact of polyphenols and polyphenol-rich dietary sources on gut microbiota composition. J. Agric. Food Chem..

[152] Man A.W., Xia N., Daiber A., Li H. (2019). The roles of gut microbiota and circadian rhythm in the cardiovascular protective effects of polyphenols. Br. J. Pharmacol..

[153] Fabris S., Momo F., Ravagnan G., Stevanato R. (2008). Antioxidant properties of resveratrol and piceid on lipid peroxidation in micelles and monolamellar liposomes. Biophys. Chem..

[154] Wang H.-L., Gao J.-P., Han Y.-L., Xu X., Wu R., Gao Y., Cui X.-H. (2015). Comparative studies of polydatin and resveratrol on mutual transformation and antioxidative effect in vivo. Phytomedicine.

[155] Du Q.-H., Peng C., Zhang H. (2013). Polydatin: A review of pharmacology and pharmacokinetics. Pharm. Biol..

[156] Cichewicz R.H., Kouzi S.A. (1998). Biotransformation of resveratrol to piceid by Bacillus cereus. J. Nat. Prod..

[157] Basholli-Salihu M., Schuster R., Mulla D., Praznik W., Viernstein H., Mueller M. (2016). Bioconversion of piceid to resveratrol by selected probiotic cell extracts. Bioprocess. Biosyst. Eng..

[158] Wang D., Zhang Z., Ju J., Wang X., Qiu W. (2011). Investigation of piceid metabolites in rat by liquid chromatography tandem mass spectrometry. J. Chromatogr. B.

[159] Bode L.M., Bunzel D., Huch M., Cho G.-S., Ruhland D., Bunzel M., Bub A., Franz C.M., Kulling S.E. (2013). In vivo and in vitro metabolism of trans-resveratrol by human gut microbiota. Am. J. Clin. Nutr..

[160] Rotches-Ribalta M., Andres-Lacueva C., Estruch R., Escribano E., Urpi-Sarda M. (2012). Pharmacokinetics of resveratrol metabolic profile in healthy humans after moderate consumption of red wine and grape extract tablets. Pharm. Res..

[161] Vogl S., Atanasov A., Binder M., Bulusu M., Zehl M., Fakhrudin N., Heiss E., Picker P., Wawrosch C., Saukel J. (2013). The herbal drug *Melampyrum pratense* L.(Koch): isolation and identification of its bioactive compounds targeting mediators of inflammation. Evid. Based Complement. Altern. Med..

[162] Most J., Penders J., Lucchesi M., Goossens G., Blaak E. (2017). Gut microbiota composition in relation to the metabolic response to 12-week combined polyphenol supplementation in overweight men and women. Eur. J. Clin. Nutr..

[163] Chen M.-L., Yi L., Zhang Y., Zhou X., Ran L., Yang J., Zhu J.-D., Zhang Q.-Y., Mi M.-T. (2016). Resveratrol attenuates trimethylamine-N-oxide (TMAO)-induced atherosclerosis by regulating TMAO synthesis and bile acid metabolism via remodeling of the gut microbiota. MBio.

[164] Zhao L., Zhang Q., Ma W., Tian F., Shen H., Zhou M. (2017). A combination of quercetin and resveratrol reduces obesity in high-fat diet-fed rats by modulation of gut microbiota. Food Funct..

[165] Qiao Y., Sun J., Xia S., Tang X., Shi Y., Le G. (2014). Effects of resveratrol on gut microbiota and fat storage in a mouse model with high-fat-induced obesity. Food Funct..

[166] Huycke M.M., Abrams V., Moore D.R. (2002). Enterococcus faecalis produces extracellular superoxide and hydrogen peroxide that damages colonic epithelial cell DNA. Carcinogenesis.

[167] Yang C., Deng Q., Xu J., Wang X., Hu C., Tang H., Huang F. (2019). Sinapic acid and resveratrol alleviate oxidative stress with modulation of gut microbiota in high-fat diet-fed rats. Food Res. Int..

[168] Sung M.M., Kim T.T., Denou E., Soltys C.-L.M., Hamza S.M., Byrne N.J., Masson G., Park H., Wishart D.S., Madsen K.L. (2017). Improved glucose homeostasis in obese mice treated with resveratrol is associated with alterations in the gut microbiome. Diabetes.

[169] Wellman A.S., Metukuri M.R., Kazgan N., Xu X., Xu Q., Ren N.S., Czopik A., Shanahan M.T., Kang A., Chen W. (2017). Intestinal epithelial sirtuin 1 regulates intestinal inflammation during aging in mice by altering the intestinal microbiota. Gastroenterology.

[170] Nohr M.K., Kroager T.P., Sanggaard K.W., Knudsen A.D., Stensballe A., Enghild J.J., Olholm J., Richelsen B., Pedersen S.B. (2016). SILAC-MS Based Characterization of LPS and Resveratrol Induced Changes in Adipocyte Proteomics—Resveratrol as Ameliorating Factor on LPS Induced Changes. PLoS ONE.

[171] Ponzo V., Soldati L., Bo S. (2014). Resveratrol: A supplementation for men or for mice?. J. Transl. Med..

[172] Bowey E., Adlercreutz H., Rowland I. (2003). Metabolism of isoflavones and lignans by the gut microflora: A study in germ-free and human flora associated rats. Food Chem. Toxicol..

[173] Bai B., Vanhoutte P.M., Wang Y. (2014). Loss-of-SIRT1 function during vascular ageing: Hyperphosphorylation mediated by cyclin-dependent kinase 5. Trends Cardiovasc. Med..

[174] Stein S., Matter C.M. (2011). Protective roles of SIRT1 in atherosclerosis. Cell Cycle.

